# Ethical Guidance for Investigational Implantable Neural Device Studies: Recommendations for Researchers and Research Ethics Committees

**DOI:** 10.1016/j.neurom.2025.12.011

**Published:** 2026-02-12

**Authors:** Odile C. van Stuijvenberg, Katherine C. Bassil, Cecile C. de Vos, Geert Geleijnse, Herke-Jan Noordmans, Marike L. D. Broekman, Rieke van der Graaf, Roland M. Bertens, Gabriel Lázaro-Muñoz, Karin R. Jongsma

**Affiliations:** Bioethics & Health Humanities, University Medical Centre Utrecht, Utrecht, The Netherlands

**Keywords:** Ethical guidelines, legal standards, neural devices, recommendations, research ethics committees

## Abstract

**Objectives:**

Clinical studies of implantable neural devices raise unique ethical and legal challenges owing to the distinctive nature of these technologies and their research contexts. Persistent issues such as inadequate posttrial arrangements, psychologic risks, and study team roles have been highlighted in the literature. This study aims to address ongoing ethical challenges in clinical studies of implantable neural devices by providing practical recommendations for researchers and research ethics committees (RECs).

**Materials and Methods:**

Based on the synthesis of a comprehensive review of academic literature, existing ethical and legal guidelines, empirical studies, and input from various stakeholders, recommendations and their practical translation for RECs and researchers have been formulated.

**Results:**

The study presents recommendations organized into five central themes: 1) the study team, 2) participant selection and recruitment, 3) informed consent processes, 4) risk-benefit assessment, and 5) posttrial responsibilities. Moreover, two procedural recommendations aim to enhance REC review processes specific to investigational implantable neural devices studies.

**Conclusions:**

These recommendations offer actionable guidance to help researchers align protocols with ethical and legal standards, and to assist RECs in anticipating and mitigating ethical risks in clinical studies involving implantable neural devices.

## INTRODUCTION

Rapid technologic advancements in the field of implantable neural devices are increasingly sparking hope for many individuals living with psychiatric, neurologic, or sensor and motor conditions. Implantable neural devices include devices that interact with the central nervous system (CNS) or the peripheral nervous system (PNS). CNS neural implants include deep brain stimulators (DBS), brain-computer interfaces (BCI), and spinal cord stimulators. PNS neural implants include vagus nerve stimulators and cochlear implants. Some implantable neural devices, such as DBS for movement disorders, have already shown significant therapeutic success across multiple neurologic conditions, becoming standard care for conditions such as Parkinson’s disease, and are under investigation for other treatment-resistant conditions.^1,2^ Others, including BCIs, are being investigated in clinical studies, increasingly indicating potential for restoring lost functions such as speech, movement, or sight.^3–5^

Clinical studies of implantable neural devices (hereafter referred to as investigational implantable neural devices IINDs, here defined as devices assessed in clinical investigation to determine their safety and/or effectiveness)—particularly those involving devices such as DBS for psychiatric conditions or BCIs—raise ethical challenges that are less prominent in clinical studies with established implantable neural devices used in standard care. For instance, the lack of posttrial arrangements in these types of studies has been discussed as an ethical concern for IIND studies, with some manufacturers ceasing operations or discontinuing support for devices, leaving participants without access or support.^6–9^ In addition, the need for long-term follow-up and support after the study ends poses specific risks and responsibilities.^10,11^ Moreover, IIND studies inevitably introduce the need for decisions about explantation, whether initiated by participants or required by researchers for medical or technical reasons, raising significant and complex ethical and legal concerns related to autonomy, consent, and harm minimization.^8,12,13^

Other ethically significant aspects of these studies include the broader psychosocial risks and benefits beyond physical effects, challenges in participant recruitment due to the highly specific needs of this research population, and the composition and role of the study team itself.^14–19^ These and broader ethical aspects of implantable neural devices also are reflected in the recent 2025 United Nations Educational, Scientific and Cultural Organization (UNESCO) Recommendations on the Ethics of Neurotechnology.^20^ Scholars also have argued that ethical obligations of justice and reciprocity are particularly strong in IIND research. These obligations are grounded in the physical invasiveness of these devices, the lack of appropriate alternative treatments and often last-resort option for people with debilitating conditions, the greater than usual uncertainty (eg, due to translational gap with animal models and very small first-in-human [FIH] studies), and the significant commitment and effort required from participants for therapeutic benefit to be achieved and maintained.^21^

These complex ethical challenges are of critical importance to both researchers and research ethics committees (RECs),^22^ given that they play a key role in ensuring the protection of study participants and ensuring scientific validity and ethical integrity of these studies. RECs in particular play a critical role in providing guidance to researchers and ensuring ethical and legal compliance with applicable legal and ethical standards, including the Declaration of Helsinki (DoH), Council for International Organizations of Medical Sciences (CIOMS) guidelines, and the European Medical Device Regulation (MDR). Despite existing ethical and legal frameworks, empirical analyses have revealed persistent and significant ethical challenges in clinical studies of implantable neural devices globally.^8,9,11,13,20,21^ Nevertheless, a significant gap remains—namely, the lack of translation from this body of literature to concrete, actionable guidance for those directly involved in conducting and reviewing IIND research: researchers and RECs, respectively.

This study aims to address that gap by offering practical recommendations for researchers designing and conducting IIND studies, and for RECs tasked with assessing these studies. These recommendations are grounded in a comprehensive analysis of the academic literature, empirical research, and feedback from stakeholders. Although the proposed recommendations are informed by European regulatory and ethical standards, the issues discussed have broader global relevance. Because the fields of IIND research and related ethical discourse continue to evolve rapidly, these recommendations are best viewed, firstly, as educational—clarifying how IIND research differs from other novel devices and procedures—and secondly, as a foundation for turning scholarly ethical discussions into practical steps for conducting responsible IIND research. Although generally applicable to all IINDs, some recommendations are device-specific (eg, BCIs), owing to differences in study duration, training requirements, and other variables; if applicable, these distinctions are highlighted throughout the article.

## MATERIALS AND METHODS

### Approach

This study presents practical guidance for RECs and researchers in the context of ethical issues in IIND studies. The work builds on prior descriptive research conducted within the Neurotechnology EXplantation: an EThical analysis (NEXT) project and the ethical work within the Innovative NeuroTEchNology for SociEty (INTENSE) project. The aim was to translate existing insights and standards into actionable recommendations, rather than to generate new ethical principles, so no formal consensus process was used. Guidance was developed by integrating findings from prior studies with established ethical and legal frameworks to support practical decision-making in RECs and research practice. The authors responsible for developing the recommendations were selected on the basis of expertise in (investigative) implantable neural device research, (bio)ethics, and REC practice, ensuring that the guidance reflects both academic debate and real-world practice.

### Data Sources and Data Analysis

Prior work comprises a systematic review analyzing the reported ethical considerations as reported in academic literature for explantation in implantable neural device studies,^13^ a systematic review focusing on the ethical implications of visual neural implants,^23^ an empirical study into the practices of RECs for assessing posttrial arrangements, psychologic harm, and explantation decisions,^24^ qualitative research into the experiences and expectations of former, current, and prospective users of implantable neural device,^25^ qualitative research into the perspectives of developers of implantable neural devices,^26^ workshops provided to researchers and RECs, and an inventory of concerns and wishes of patient representatives for visual neural implant studies. These findings were cross-checked with existing relevant guidelines and legislation referred to in these recommendations, including the European Union (EU) MDR Regulation (EU) 2017/745 (in this article referred to as “MDR”), the 2024 Revised Declaration of Helsinki (in this article referred to as “DoH”), the 2016 CIOMS International Ethical Guidelines for Health-related Research Involving Humans (in this article referred to as “CIOMS”), and the 2025 UNESCO Recommendations on the Ethics of Neurotechnology (in this article referred to as “UNESCO”).

### Synthesis

The recommendations are centered around five themes of key ethical considerations for research with human participants, including 1) the study team, 2) selection of participants, 3) information and consent, 4) benefits and risks, and 5) posttrial arrangements. The themes selected follow the structure and themes addressed in research protocols, allowing the recommendations to align closely with existing REC review processes and to be applicable across the different stages of study design and review. For the formulation of recommendations, we implemented the following rationale: Recommendations following directly from legal provisions are formulated as “must”; those following from ethical guidelines have been formulated as “should,” and those following from ethical academic literature as “it is recommended.” Each recommendation is supported by reasons “why” this recommendation is deemed important and “how” the recommendations can be operationalized.

## RESULTS: RECOMMENDATIONS FOR RESEARCHERS AND RECs

This study outlines a set of recommendations that include both relevant provisions already established in existing legal frameworks and ethical guidelines, for which empirical studies have indicated the need for an increased or renewed attention when designing and assessing IIND studies, in addition to further recommendations and provisions to improve the depth and width of REC assessment of IIND studies.

### The Study Team

The composition of the study team in IIND research warrants careful consideration, given the complex and often conditionspecific needs of the participants in the study. Moreover, the various physical, functional, and emotional impacts of IINDs introduce the need for specific types of support—such as clinical or ethical guidance, and psychologic and emotional support. Therefore, different skills and expertise should be reflected in the study team to ensure that patient well-being is considered throughout the study. RECs should assess whether all the required skills are present in the study team before starting the study.

The Study Team Should Have Sufficient Time and Resources to Address Potential Participants’ Communication Needs

WHY: Participants in IIND studies often have sensory, neurologic, or psychiatric impairments, complicating standard communication and consent procedures,^13^ as also noted in UNESCO recommendation 45 and DoH Article 26^20,27^ (Information and Consent section).

HOW: Researchers should allocate time and possess the skills to adapt communication, using tailored information and specialist support to ensure participant understanding and engagement.^28–30^ Insights from patients or patient representatives could be especially helpful when designing communication strategies and consent processes for these studies, for instance, by recommending ways an informed decision can be facilitated by adequate communication aids or services, such as sign language interpretation or consent forms in braille.^28,31^

It Is Recommended That the Study Team Comprises Multidisciplinary Expertise

WHY: IINDs can benefit participants but may simultaneously cause emotional distress and changes to sense of self, and can challenge existing relationships, requiring monitoring and support from the study team.^11,32–34^ Moreover, investigational IIND studies can be demanding, with some studies requiring extended daily laboratory sessions for long periods.^3,21,35^

HOW: To address the diverse implications and burdens of explorative IIND studies, a multidisciplinary team is essential for risk assessment, monitoring dissent, and supporting participants.^36,37^ It is recommended that this team includes neurosurgeons skilled at safe implantation and explantation, psychologists to manage mental health risks, and technical experts for device design, development, maintenance, and rehabilitation, particularly when the device does not perform as intended. These experts contribute not only to the maintenance of the technology but also to refining it on the basis of empirical findings. In addition, incorporating experiential knowledge from past participants and patient communities can further inform researchers and improve study design, as also outlined in UNESCO recommendation 62.^20^

It Is Recommended That Measures to Preserve Continuity of the Study Team Are Embedded in the Study Protocol

WHY: Discontinuity of the team has been considered burdensome or a cause for worries for implantable neural device users; continuity also is deemed highly important by developers of IINDs.^26^

HOW: It is recommended that in the event of changes in the study team, key skills and support roles are replaced, with any transitions clearly communicated to participants—particularly when team members provide psychologic support (See section above about sufficient time and resources). For IINDs that remain implanted after trial, it is recommended that a designated point of contact is available to assist participants in case of device-related issues. Anticipated future changes in team composition, strategies for replacing expertise, and measures to ensure continuity of care should be outlined in the study protocol.

### Recruitment and Selection of Participants

IIND studies typically include only a small number of participants. Given the lack of effective alternative treatments for these conditions and the demandingness of IINDs studies for sensory or motor conditions (eg, visual neural implants or speech-BCIs), recruitment and selection of study participants require increased attention.

This is particularly important because the effects of these IINDs may be temporary, given some implants may lose function over time (eg, visual implants) and some devices may be explanted at the end of the study or removed owing to unforeseen complications. Recognizing this temporariness is important in the management of participant expectations during the planning of a study.

Exclusion Criteria Should Be Justified, and It Is Recommended That Selection Is Motivated

WHY: IIND research typically includes populations with sensory or movement impairments (eg, people with blindness or paralysis) or with increased risk of mental vulnerabilities or neurologic disorders (eg, people with depression or chronic pain). Exclusion from research studies should not be based a priori on comorbid diagnoses such as psychiatric conditions and/or owing to high rates of explantation in certain patient groups.^38,39^ Categorical exclusion, particularly of groups requiring special protection, should be clearly justified, given it can contribute to or worsen existing health disparities, as outlined in CIOMS guideline 3 and DoH article 19.^27,40^

HOW: RECs should assess whether the harms of exclusion of certain groups a priori outweighs the harms of inclusion for patients who are deemed vulnerable. Participants eligible but not selected for clinical studies may appreciate receiving information from the researchers about the reasons for exclusion from the study.

The Selection of Participants Must Safeguard the Capacity of Potential Participants to Bear the Burdens Associated With Participating in the Study

WHY: Clinical IIND studies may impose a significant burden on study participants, owing to their intensity and duration.^21^ Moreover, implantable BCI research can involve extensive training for the use of a device, an aspect participants may not fully appreciate at study onset.^36^ In terms of legal requirements, the MDR (article 62, paragraph [par.] 4 under h) stipulates that studies may only be undertaken with participants whose rights to physical and mental integrity are safeguarded.^41^ This affects the way in which the ability of individual participants to handle the burden is assessed, given including participants who clearly cannot do so can easily lead to a violation of these rights.

HOW: Selection of participants should include an individual assessment (including, eg, psychologic assessment) of their expected capacity to handle the burden of study participation and should not categorically exclude groups on the basis of group characteristics (See section above about the justification of exclusion criteria …).

It Is Recommended That Selection Criteria Include Emotional Tolerance of Participants

WHY: IIND studies, especially those focusing on BCI for sensor or motor impairment, may lack therapeutic effectiveness, may have temporary therapeutic effects, or may require explantation at the end of the study, which can cause significant emotional and psychologic effects.^11^

HOW: It is recommended that FIH studies prioritize participants who are comfortable with their current condition, do not expect direct therapeutic benefit, and are emotionally equipped to manage uncertainty, to minimize potential disappointment and psychologic harm. Assessment of emotional tolerance should be integrated into the selection process and ideally conducted by an expert independent evaluator (ie, a mental health expert with no relevant conflict of interest with the study).

It Is Recommended to Assess Whether Potential Participants Have Sufficient Support for Study Participation

WHY: Study participation for IINDs often depends on adequate external support. Care partners frequently play a vital role, particularly for participants with severe disabilities such as locked-in syndrome.^36,42–47^ Participation may increase the burden on care partners, introducing risks such as fatigue-related compromises in caregiving quality. Other participants may lack such a network altogether.

HOW: It is recommended that the research team assesses whether a care partner is available, able, and willing to take on required study tasks and participant support. It is recommended that care partners also be asked for consent,^36^ and/or equally receive (posttrial) psychologic support and that their perspectives and consent considerations receive careful attention.^43^ If no study partner is available, the study team could provide additional support to arrange logistics, care, and psychologic support. It is recommended to assess which support is needed by all participants and care partners.

### Information and Consent

Information about IIND studies can be complex, and participants face important decisions about both the outset and end of the study. For instance, the end of the study involves decisions such as options to keep the device and continue its use, deactivation of the implanted device, or partial or complete explantation of the device.

Information Must Be Clear, Regularly Revisited, and Adapted to Participants’ Evolving Understanding and Needs

WHY: Information in IIND studies is often highly technical and difficult to comprehend.^36^ Studies of DBS reveal that participants may have difficulty retaining key posttrial details, such as device maintenance or explantation.^7,15,48^ Moreover, participants may, in their initial consent, prioritize short-term benefits including symptom relief, with limited awareness of long-term consequences.^7^ Furthermore, participants’ perspectives on the benefits, implications, and posttrial preferences can evolve, influenced by changes in well-being or sense of self due to the implant.^11,13^ To support autonomous decision-making, information should be clearly communicated, repeated throughout the study to prevent overload, and tailored to participants’ evolving needs (section titled The Study Team Should …), as also outlined in DoH article 26 and UNESCO recommendations 45, 46, and 108.^20,27^ This also is a challenging legal requirement, as set down in both European and national legislative frameworks regulating medical trials, including MDR article 62 (par. 4 under f) and article 63.^41^ Crucially, at all times, participants (or their legal representatives) must understand the nature, objectives, benefits, implications, risks, and inconveniences of the study. Moreover, information concerning the participant’s rights, in addition to treatment alternatives, duration of the study, and consequences of discontinuation of (participation in) the study, must be discussed clearly and comprehensively.

HOW: Researchers should develop a more iterative consent procedure, with regular communication and verification of understanding. Sufficient time should be allocated for initial consent, followed by frequent information check-ins during the study, to ensure participants remain informed about critical decisions, including explantation options.^7^ This approach allows participants to revisit, revise, or reconsent to earlier decisions.^7,36,37^ Several critical points for reconsent can be predefined, such as at inclusion and after successful screening. Yet other critical points, such as major situational changes to participants (eg, leaving of a care partner) or changes to the character or mental state of the participant, may be less easily predefined. Still, in individual studies, input from the research team (including psychologic experts) can assist in identifying moments that could influence (re)consent.

It Is Recommended That Researchers Ensure Realistic Expectations of Study Participants About Anticipated Benefits, Risks, Burdens, Participation Requirements, and Responsibilities

WHY: IIND technologies speak to the imagination, making them appealing, but also pose a risk for unrealistic expectations of potential users. Managing participant expectations is crucial in IIND studies to prevent overpromising and avoid conveying unrealistic prospects regarding the intervention’s benefits.^23,26,36^ Moreover, it is recommended that participants be informed about ongoing responsibilities in long-term studies requiring sustained engagement, such as regular training to maintain device efficacy and other research activities.^3,21,23^ Clear communication of the study’s goals, demands, and realistic outcomes helps mitigate therapeutic misconception, misplaced hope, or despair, while distinguishing these from legitimate treatment search fatigue,^49^ and prevents false hope reinforced by media-driven narratives about neurotechnology.^36,50^

HOW: It is recommended that participants are informed accurately about the study’s goals, burden, and realistic outcomes expected from the intervention. A multidisciplinary research team can help effectively communicate these risks and expectations (See the sections above about Sufficient time and Resources and multi-disciplinary expertise in the study team).^36^ Researchers should clearly outline expectations for adherence to study protocols, training requirements, and device maintenance and care. In addition, involving an independent mentor, patient representative (eg, patient’s caregiver), or professional confidant in the research team can assist participants in understanding the information and minimizing unrealistic expectations.

Informed Consent Procedures Must Include Information About the Ending of the Study, Including Plans and Possibilities for Explantation and Posttrial Arrangements

WHY: IIND studies can end in different ways because the device may be explanted or remain implanted. Adequate information about relevant parts of the study, including the ending of the study and posttrial arrangements, is essential for participants. This also is reflected in CIOMS guideline 6, DoH article 26, and MDR article 63 (par. 2 under a iv).^27,40,41^ Access to IINDs after the study ends, particularly for BCIs, has not always been guaranteed, owing to limited posttrial options. In other cases, explantation of the investigational device may be planned, which carries its own risks (See also section below about future eligibility).^11,13,23^ Providing information about posttrial arrangements before the study begins, to be revisited throughout the study, is key to meaningful informed consent.^7,16,17,24,51^

HOW: Before the start of investigational IIND studies, participants should be informed about a number of topics including 1) the conditions for posttrial care; 2) the potential medical, psychologic, and financial consequences including costs related to the removal and rehabilitation;^17,52^ and 3) the impact of explantation or remaining implanted on eligibility for future implantable neural device(s) (and IIND studies) (See sections below regarding financial risks of research and rights of study participants in case of bancruptcy). This information should be repeated regularly (as outlined in section titled Participants Must Maintain …) to ensure participants remain well-informed, including topics such as the possibility of early study termination due to ineffectiveness (section titled It Is Recommended to Assess …), safety concerns,^7,9^ or financial challenges.^8^

Participants Must Maintain the Right to Withdraw From the Study at Any Time Without Reprisal, Even When this Necessarily Involves (a Decision on) Explantation

WHY: Participants in IIND studies have the right to withdraw at any time, as set out in MDR article 62 (par. 5) and DoH article 26,^27,42^ which may include requesting or refusing explantation at withdrawal. Participants may have personal, emotional, or psychologic reasons for wanting to withdraw from a study or for refusing explantation at withdrawal,^13^ such as experiencing changes in their sense of self due to the implant.^11^ Therefore, participants should be informed about the right to, and implications of, withdrawal from a study and the associated risks. However, participants are not obligated to justify their withdrawal.

HOW: The right to withdraw should be respected and clearly communicated to participants. Moreover, the implications of withdrawal from the study for the use and potential explantation of the device in addition to the associated medical care should be made clear to participants. This includes ensuring that participants understand the potential distress or harm associated with explantation at withdrawal from the study. Participants cannot be forced to undergo surgery for the explantation of the device because that would imply a form of coercion and may violate the principle of nonmaleficence. At the same time, explantation can be necessary to avoid severe medical risks and complications. Hence, refusal of explantation from the start may be considered an exclusion criterion. To ensure transparency and respect for participants’ autonomy, exclusion criteria should be communicated clearly to prevent potential misunderstandings. Involving psychologic expertise to support understanding and address any emotional or psychologic factors influencing the decision to withdraw is recommended.

Voluntariness of Consent Must Be Ensured

WHY: Voluntariness of consent may be compromised in small and active IIND studies when participants depend on treating physicians or maintain close collaboration with the research team, particularly in motor and sensory implant studies. Factors such as team dependency, as described in CIOMS guideline 9 and DoH article 27,^27,41^ fluctuating decision-making capacity in patients with neurologic or psychiatric conditions,^53^ as described in UNESCO recommendations 46 and 108,^20^ and lack of alternative treatments can further affect voluntariness.^22^ RECs must assess whether study relationships or conditions undermine voluntary consent, including consent for explantation or posttrial decisions, and ensure participants’ decision-making capacity is adequately evaluated.^22,53^ Voluntariness of consent and the lack of undue influence also are legal requirements on the basis of the MDR article 62 (par. 4 under k).^42^

HOW: To reduce dependency, individuals involved in a participant’s therapeutic care (eg, treating physicians, nurses) should not be part of the consent process.^22,27,40,54,55^ Existing care relationships with the research team should be disclosed and evaluated by RECs. For participants with mental health conditions, involvement of an independent third party, such as a family member or clinical psychologist, can support assessment of decision-making capacity.^53^ Consent should be obtained at an appropriate time, accounting for fluctuating competence or emotional distress, and care partners may be involved for proxy consent when necessary.^56^ Language and communication must be carefully managed to avoid subtle coercion, particularly when treatment alternatives are limited.

### Benefits and Risks

Risk-benefit assessment of IIND studies should include a wide array of risks and benefits, including physical, privacy, psychologic, and financial aspects, in addition to long-term implications of study participation and device implantation and explantation.

#### Benefits

It Is Recommended to Assess to What Extent Clinical End Points of the Study Correspond to Benefit for Participants.

WHY: Reaching study end points does not always align with patient satisfaction.^13,21,57,58^ For instance, patients may experience meaningful benefits and satisfaction even if the clinical end point is not reached.^58,59^ Conversely, some study end points may not be sufficient to realize patient benefit (eg, reducing frequency of epileptic seizures may not significantly change the quality of life of patients).^60^ In such cases, patient dissatisfaction may cause patients to prefer explantation because the device was not considered meaningful.^13^

HOW: It is recommended that patient-centered outcome measures are included as study end points (eg, about quality of life or functional restoration). In addition, patients can be included in the design of the study to shape patient-relevant outcomes, and researchers are encouraged to report on patient-centered outcomes as part of the study.

#### Physical/Mechanical Risk and Harm

Quality and Stability of the Material, Device, and Software Must Be Part of the Risk Assessment.

WHY: Innovative materials and software are being developed for IINDs. In line with the MDR,^42^ and International Organization for Standardization (ISO) 14708-1 (for active implantable medical devices) in addition to ISO 10993 (for biological evaluation),^61^ research protocols must include detailed preclinical data on the stability and biocompatibility of the material and the device. Furthermore, IINDs may lose functionality over time owing to material degradation or software issues,^12,23,36^ meaning that long-term effects of these devices that remain implanted are uncertain or may pose risks to participants.

HOW: Preclinical data on material stability, especially for IINDs intended to remain implanted after the end of the study, should be reviewed by RECs to assess physical risks of implantation, notwithstanding the legal obligations that rest with manufacturers. Researchers are encouraged to collect long-term safety data on the stability of IINDs,^12,36,62,63^ as also outlined in UNESCO recommendation 107.^20^ Posttrial continued access to IINDs may offer an important opportunity for gathering such long-term data.

RECs Must Assess the Risks of Explantation and of Long-Term Implantation.

WHY: Both the long-term maintenance of IINDs that remain implanted and their explantation involve risks. IINDs must be tested in accordance with the MDR and ISO 10993 to prevent toxicity, biological incompatibility, infections, migration of the device (mostly in the early weeks post implantation), and related device-specific side effects.^41,61^ Explantation is paired with mechanical risk and harm, including scar tissue formation and risks for psychologic harm.^11,12,23^

HOW: A comprehensive risk assessment of explantation (physical, psychologic, financial, and privacy) and risks of keeping the device implanted should be included for REC review. This assessment should also inform posttrial arrangements of the study (See the section below titled Posttrial Arrangements). Researchers should implement measures to minimize the risks and burdens, and continuously monitor, assess, and document the risks and burden, in accordance with DoH article 17.^27^

Risks for Future Eligibility of IIND Studies and Access to Implantable Neural Device Treatment Should Be Assessed.

WHY: Owing to the rapid technologic advancements in the neurotechnology field, investigative devices, especially BCIs, become rapidly obsolete. This poses risks for both study participants who remain implanted with these devices and those who are explanted. Users of experimental devices that remain implanted after trial may be left without functioning devices, support, or spare parts, and without possibilities to receive newer implants.^6,64^ Furthermore, explantation after the study ends may mean that participants are no longer eligible candidates for other neural device studies or treatment by later-generation implantable neural devices because of scar tissue formation during implantation or explantation.

HOW: It is recommended that RECs assess risks related to 1) availability of spare parts and other support (for those who remain implanted), 2) the compatibility of the implanted device with newer generation devices (for those who remain implanted), and 3) alternative treatment options for those who are explanted. Promoting the use of implanted hardware that is, or could be, compatible with newer devices (eg, standardized or “gold standard” hardware) could increase chances of future eligibility for newer implants, also in line with UNESCO recommendation 106.^20^ These risks must be clearly communicated to potential participants before and after the study (See also section about information about the ending of the study).

It Is Recommended to Assess Risks of Implantation for Physical Harms, Such as a Lack of Compatibility With Diagnostic and Other Health Interventions.

WHY: Incompatibility of (remaining) implanted hardware with other technology can have long-term implications for (former) participants in a study. IINDs may interfere with magnetic resonance imaging or other diagnostics and treatments (eg, external defibrillation).^65,66^ During participation, and after trial when implanted hardware is not removed, patients may lose access to such diagnostics and treatments,^21^ which could lead to (serious) health risks, especially as participants age or their condition worsens.

HOW: It is recommended that the incompatibility of IINDs with (future) diagnostics and treatments, in addition to the associated risks, is assessed and proportionate to the foreseen benefits of study participation.^66^ It is recommended that (former) participants, and their current and future health care providers, are adequately informed about technologic (in)compatibility risks for their future care. It also is recommended that participants be reminded of incompatibility risks when leaving the study and that compatibility risks be noted in the electronic patient file.

#### Psychological Risks and Harm

Psychological Impact Must Be Monitored, Assessed, and Mitigated During the Study.

WHY: Currently, relatively little is known about the psychological impact of IINDs on participants. Changes in cognition, sense of self, and emotional well-being due to IINDs have been reported in the literature.^12,24,67^ Although such impacts may be experienced as negative and undesirable (eg, as intrusive and disruptive),^68^ positive experiences in sense of self, mood, and behavior changes also are reported.^11,57^

HOW: It is recommended that psychological well-being is assessed and compared before implantation, during implantation (with device switched on and with device switched off), and at the end of the study, to identify psychologic impacts. During the study, well-being must be continuously monitored, in accordance with MDR article 62 (par. 4 under I), and as reflected in UNESCO recommendations 55, 104, and 113.^20,41^ Mitigation strategies for psychological risks and harms (eg, undesirable changes) include the involvement of psychologists during and after the study and when applicable, posttrial support (See sections below about the availability of posttrial arrangements and responsiveness of Post-trial Arrangements). In cases when IINDs are applied for psychiatric conditions and a specific type of psychologic or behavioral change is the desirable outcome, the resulting nature of a(n) (un)desirable change should be clearly defined at the start of the study.

#### Privacy

Undue Privacy Risks to Personal Data of Participants and Bystanders Must Be Prevented.

WHY: IINDs can record, transmit, or stimulate brain activity that when decoded is considered sensitive so should be paired with additional legal protection.^69^ Furthermore, IINDs may record visual or auditory data of the participants in public spaces, meaning that sensitive and privacy-sensitive data of themselves and others may be collected, as defined in the EU General Data Protection Regulation article 5 and 9,^70^ and MDR article 62 (par. 4 under h).^42^ The need to carefully handle and protect brain data to ensure privacy protection also is outlined in DoH article 24 and UNESCO recommendations 50, 87, and 91.^20,27^ Moreover, the growing academic literature and discussion on the need for increased protection of neural data further supports this recommendation.^69^

HOW: Careful assessment of data collected by IINDs and their alignment with relevant privacy regulations must be in place to protect the privacy of participants and bystanders.

It Is Recommended That *Support* and Risk-Mitigation Is in Place for Personal Privacy Risks Such as Intrusive Media Attention.

WHY: Users of previous neurotechnology have encountered negative experiences in the form of intrusive attention by journalists and the media.^25^ These users were easily identifiable because their devices were exposed and visible to bystanders. The same risk may be applicable for IINDs.

HOW: Sufficient media-related support and protection are recommended for all participants. It is recommended that measures are taken to prevent harm due to intrusive media by involving appropriate expertise (eg, of media trainers) and are tailored to participants’ needs because individual participants’ preferences regarding media attention can vary.^25,26^

#### Financial Risks

Financial Risks of Research Participation Should Be Assessed.

WHY: Device maintenance and explantation are costly and require sufficient funding,^18^ presenting significant challenges for sponsors, hospitals, and patients. Given the dependency, vulnerabilities, and impairments of some of the target populations for IINDs, this requires careful assessment by RECs.

HOW: The financial impacts of explantation (during and after the study), device maintenance, and access to psychologic support, and risks that these costs will be for study participants, should be assessed by RECs and should be mitigated before the start of the study, as outlined in DoH article 34.^27^ RECs should assess the appropriateness of the proposed period of medical and psychologic care covered by the sponsor or hospital after the study ends (section titled Posttrial Arrangements).

Rights of Study Participants Must Be Protected in Case of Bankruptcy of the Manufacturer.

WHY: In the past, several neurotechnology companies have gone bankrupt, without any (posttrial) arrangements for users and (former) participants in a study, meaning there was no support, spare parts, or accountable party available.^9,11,71–73^ A lack of contingency plans and posttrial arrangements means that participants in the IIND study face financial risks should they choose to keep the device.^8^ Article 69 of the MDR expressly stipulates that member states shall ensure that systems for compensation for any damage experienced by a subject resulting from participation in a clinical investigation conducted on their territory are in place in the form of insurance, a guarantee, or a similar arrangement.^42^ It is recommended that the risk of bankruptcy and resulting financial damages for participants are explicitly covered by these insurance arrangements, as outlined in UNESCO recommendation 111.^20^

HOW: Financial and medical risks arising from bankruptcy should be considered in the RECs’ risk assessment of the study, with particular attention from legal experts within the committee. These risks also should be addressed in the informed consent process and posttrial arrangements (See the section below mitigation plans for financial risks). Specifically, RECs should evaluate and ensure that during recruitment, participants are informed of the potential financial risks of manufacturers during the trial and their rights as participants for continued access and support. Researchers should address these in the informed consent process and posttrial arrangements. Institutional mitigation planning for such risks is addressed under the section about mitigation of financial risks.

#### Posttrial Arrangements

IIND studies introduce additional challenges and requirements for the posttrial period, compared with pharmaceutical studies and other non-IIND studies. The ending of IIND studies inevitably requires decisions to be made on the fate of the implant (ie, whether it should be explanted, remain implanted and turned off, or remain turned on), which can have significant short- and long-term consequences for participants in the posttrial period. Participants who experienced benefit during the study may wish to continue to use the device after the study ends, and participants who are explanted at the end of the study may still need psychologic support related to study participation. Posttrial arrangements are essential for the responsible conduct of studies with IINDs and can include different types of support in addition to mechanisms for continued access to the investigational devices.

Posttrial Arrangements Should Be Available to All Participants of the Study, Regardless of Their Individual Trial Outcomes.

WHY: Posttrial arrangements, including provisions for continued access, are important for IIND studies because these devices can affect basic capabilities such as communication or independent functioning. Former and current participants often cannot access these devices within standard health care systems owing to their investigational character and high costs, and absence of available alternatives with clinically effective alternatives, as set out in CIOMS guideline 6.^41^

HOW: Posttrial arrangements (eg, psychologic support) should be available for all participants in a study, including those who have been explanted during or after ending the study. These arrangements should be made in advance of the study, in line with CIOMS guideline 6 and DoH article 34,^27,41^ and are recommended to include the possibility for continued access, posttrial medical and psychologic care for those implanted and explanted during the study, and explantation options.^8,10,11,13,16^ These arrangements are recommended to be based on both individual participant needs (See the section below about responsiveness of posttrial arrangements) and evaluation of (long-term) risks and benefits. RECs are recommended to review financial arrangements for the posttrial period, including costs for explantation operation, revalidation, and medical care (including psychologic support).

It Is Recommended That Posttrial Arrangements Be Responsive to Individual Participant Needs.

WHY: The type(s) of posttrial care needs may differ among individual participants on the basis of, for instance, their level of experienced benefits and harms during and after the study. For instance, although some participants may require higher levels of psychologic support related to study participation, others may only require a limited level (or none).

HOW: It is recommended that posttrial arrangements are tailored to individual participant needs and preferences, provided they are directly related to (past) study participation. The close collaboration between the research team and participants can facilitate the individualization of posttrial arrangements according to each participant’s needs. If medical and psychologic care is required in the posttrial period because of (the) medical condition(s) or events other than study participation, researchers are recommended to refer former participants to medical care as a transition to care after research requirement consistent with CIOMS guideline 6.^41^ Notably, in some instances, care partners also may be considered participants in these studies and may have individual posttrial care needs (eg, psychologic support) that warrant attention.

It Is Recommended to Offer Posttrial Access to the IIND for All Participants Experiencing Benefits.

WHY: IINDs may come with various benefits (See above the section about clinical endpoints). In addition to therapeutic or functional benefits, IINDs also may come with other (nonfunctional) benefits, such as the intrinsic value from partial sensory restoration^35,74^ and the sense of regained agency.^11^ Withdrawing benefits of the IIND through explantation is undesirable, particularly when participants have no other way to gain these benefits, for instance, if explantation makes them ineligible for other implants.

HOW: It is recommended to have posttrial arrangements including continued access for all participants experiencing benefits, including nonfunctional benefits of the technology.^21^

Participants Must Have a Choice About (Partial) Explantation of the Device During and at the End of the Study.

WHY: Forced explantation is not ethically or legally acceptable, especially when a participant benefits from the device and wants to continue its use, given this infringes on fundamental rights including self-determination and the right to life,^11,17,64,75,76^ and is in conflict with DoH article 9, CIOMS article 1 and 9, the 2016 European Convention on Human Rights, MDR article 62 (par. 4 under h) and UNESCO recommendation 111.^20,27,41,42,77^

Participants should always retain the right to withdraw from the study at any time. This may include requesting full or partial explantation or refusing surgery to remove the device (See section above about right to withdraw).

HOW: It is recommended that explantation decisions are handled on a case-by-case basis, considering the individual’s preferences, the evolving risk-benefit profile over time, and the specific nature of the device. It is recommended to consider explantation as a distinct phase of the study that requires renewed informed consent,^64,76^ and in some jurisdictions, this may be a legal requirement.

To Ensure Appropriate Posttrial Arrangements, It Is Recommended That Mitigation Plans are Prepared for Financial Risks Including Bankruptcy of Manufacturers.

WHY: IIND studies are costly and come with financial risks, also for the sponsor, including risks of bankruptcy, which may lead to abandonment of the technology and discontinuation of the study. Earlier cases of discontinuation of IIND studies (for long-term use) due to financial issues of the sponsor (including bankruptcy) have been reported,^9,64^ leaving participants without replacement parts of functioning devices, software updates, and additional support (See sections above about Financial risks of research participation and Rights of study participants in case of bankruptcy).

HOW: A study should not be initiated if insufficient economic resources impede participants from exercising their rights, including the right to leave the trial and have the implant removed,^18^ or to refuse explantation and retain the implant. It is recommended to assess contingency plans that ensure that studies have sufficient financial resources for posttrial arrangements for all participants (See section above about availability of Posttrial Arrangements) before the start of a trial. Mitigation plans for financial risks are part of the posttrial arrangements. For participants who remain implanted and continue to use the device after trial, this includes availability of spare parts and costs and required knowledge for reparation and replacement (of both hardware and software components) to prevent these participants being left without functioning devices after trial owing to bankruptcy.^6,64^ For participants who remain implanted but no longer use the device, this includes costs and required knowledge for continued care, and for those who are explanted, this could include costs for psychologic care (See also the section above about availability of Posttrial Arrangements). Formats for posttrial arrangements addressing financial risks have been proposed in recent literature, including compensation programs^78^ and allocated research funds.^51^

Long-Term Data Efficacy and Safety Data Must Be Collected After Ending the Study.

WHY: Much remains unknown about long-term benefits and risks of implanted neural devices, especially when innovative designs and materials for electrodes are used.^23^ Follow-up of efficacy and safety data after trial and post market is essential for updated benefit-risk assessment of IINDs, as outlined in MDR article 83 and UNESCO recommendation 104.^20,42^ For CE-marked devices, this obligation falls under the manufacturer’s postmarket surveillance. For investigational devices that remain implanted after a study, sponsors of clinical institutions may need to assume this responsibility.

HOW: Researchers should be encouraged to continue monitoring former participants in a study to ensure patient-wellbeing and gather data on long-term benefit and risks. For instance, 1) researchers could coordinate follow-up with participants’ treating clinicians to ensure continued oversight of physical and psychologic health while collecting standardized data for research purposes; 2) clear pathways could be implemented for participants to report any unexpected effects or complications after study completion, ensuring that these events inform ongoing risk-benefit evaluations; and 3) researchers could periodically update RECs or regulatory bodies with aggregated follow-up data to allow refinement of study protocols, informed consent materials, and posttrial care recommendations.

Additional Procedural Recommendations for IIND Studies

In addition to the detailed recommendations listed above, we formulate two procedural recommendations for RECs assessing these studies to do justice to the specific types of knowledge necessary to comprehensively assess these types of IIND studies.

First, a consultation with neuroscientific researchers or neuromodulation medical specialists is beneficial when specific technical or scientific knowledge is needed for assessing such IIND protocols. Such consultation can contribute to ensuring that risk assessments are based on and tailored to the specific risks posed by the specific device, type of surgery, and location of the implant.

Second, input from patients from the specific study populations is highly recommended when drafting and assessing IIND research protocols, as set out in UNESCO recommendation 62,^20^ given these populations may face specific sensory or motor impairments or psychologic conditions that should be considered when designing the study. Engaging patients can help to formulate conditions to mitigate the burden of these studies, to address objections of patients, and to enable meaningful communication with potential participants (eg, braille, or sign language experts).^79^ This will not only inform better REC assessments but also will be beneficial in terms of informing patient decisions, for retaining participants in studies, and can help to reach beneficial outcomes of study participants.^80^

## CONCLUSION

The academic literature has reported on several risks and ethical challenges for IIND studies. The analysis of these risks and challenges provides a valuable starting point to develop strategies for the assessment and development of future IIND studies. These include aspects related to the composition of the study team, recruitment and selection of participants, informed consent considerations, study-associated risks and benefits, and posttrial arrangements.

The list of recommendations draws attention to overarching issues that require additional attention from academic scholars, researchers, and RECs. First, it becomes clear from our study that only little formal ethical and legal guidance is available for the requirements regarding the recruitment and selection of participants that addresses the specific challenges of IIND studies, and that existing guidance falls short of ensuring that the needs of participants in IIND studies are met in terms of the constitution of the study team and posttrial arrangements. Because many of these recommendations currently lack grounding in ethical and legal guidelines, regulators and guideline developers should recognize the need for more formalized guidance to mitigate potential harms of IIND studies. At the same time, many of the informed consent recommendations we have formulated in addition to some of the posttrial arrangement recommendations, are grounded in ethical or legal guidelines, yet empirical and ethical debate has indicated that these standards are not sufficiently met in some IIND studies. Informed consent and posttrial arrangement should therefore receive additional attention from researchers and RECs when drafting and assessing IIND studies, respectively.

Second, the conditions for IIND studies are influenced by underlying differences between various types of implants because each is associated with distinct risks and requires specific provisions for participant safety, informed consent, and long-term management. These vary, for example, in terms of intensive training, ongoing technical support, and emotional adjustment for IINDs that are foreseen for long-term use. RECs should consider these variations, given tailoring ethical review to the nature of the implant is essential to ensure proportionate protections and to support responsible innovation in this rapidly evolving field.

Third, IIND studies pose both short- and long-term risks that also are tied to the required implantation and potential explantation of the device. Long-term risks may include complications related to device malfunction, explantation challenges, psychologic effects, and interactions with future medical care. Inadequate consideration or communication of such risks may not only compromise informed consent but also affect participants’ eligibility for subsequent treatments or clinical studies. Specifically, the communication about and weighing of long-term risks are therefore in need of additional attention.

Fourth, the financial risks associated with these studies present significant challenges for sponsors, hospitals, and patients. These risks not only affect the ethical aspects of such studies, as previously discussed, but also may prevent stakeholders from following through and thus ultimately hindering scientific advancement. To address this challenge, formal mechanisms to mitigate financial risk, such as dedicated insurance schemes for sponsor bankruptcy, should be further evaluated in collaboration with researchers, sponsors, insurance companies, and developers of these implantable technologies.

### Strengths and Limitations

These recommendations are based on empirical insights, academic literature review, and expert discussions, which offer valuable depth and context to the ethical challenges associated with IIND research. Some of the identified ethical concerns may be of broader relevance and translate to noninvasive brain interventions such as repetitive transcranial magnetic stimulation and high-intensity focused ultrasound. The absence of systematic input from a broader range of research ethics committees and policy makers limits the generalizability of the findings. As a result, the recommendations may not fully capture the diversity of institutional practices or regulatory constraints across different settings. The European context was taken as a starting point, meaning that these recommendations may not translate integrally to other regions and jurisdictions. Moreover, the perspectives represented may reflect specific disciplinary or normative positions, rather than a formal consensus. Broader stakeholder engagement, including input from regulatory bodies, clinicians, and patient communities, would be fruitful future avenues to validate and refine these insights for practical application.

As implantable neural device technologies continue to evolve, so must the frameworks and procedural tools used to assess them. The recommendations formulated in this article should be considered a recalibrated starting point for researchers to align their research protocols and arrangements with ethical and legal norms and provisional ethical insights, and for RECs to assess the protocols to mitigate risks and anticipate ethical challenges. Alignment with these norms will have benefits for research institutes, hospitals, and individual researchers. By ensuring that studies investigating the therapeutic potential of IINDs are ethically aligned, the benefits can be maximized while minimizing risks and harm to patients and participants in IIND studies.

## COMMENT

This study presents a list of recommendations to help formulate strategies for the assessment and development of studies involving implantable neural devices, which often present unique ethical and legal challenges.

Owing to the distinctive nature of these technologies, the list of recommendations aims to help researchers align study protocols with ethical and legal standards, and to assist research ethics committees in anticipating and mitigating ethical risks and harm to patients and study participants. The recommendations are best viewed as educational, with the aim of turning scholarly ethical discussions into practical steps for conducting responsible implantable neural device research.

Kliment Gatzinsky, MD, PhD

Gothenburg, Sweden
